# An unusual example of hereditary multiple exostoses: a case report and review of the literature

**DOI:** 10.1186/s12891-021-03967-6

**Published:** 2021-01-21

**Authors:** Rebecca Chilvers, James A. Gallagher, Nathan Jeffery, Alistair P. Bond

**Affiliations:** 1 Human Anatomy Resource Centre, University of Liverpool, Sherrington Building, Ashton Street, Liverpool, L69 3GE UK; 2 Department of Musculoskeletal and Ageing Science, Institute of Life Course & Medical Sciences, University of Liverpool, Liverpool, UK

**Keywords:** Hereditary multiple Exostoses, Diaphyseal aclasis, Osteochondroma, Enchondroma, Synostosis, Metachondromatosis

## Abstract

**Background:**

Hereditary multiple exostoses (HME) is a rare skeletal disorder characterised by a widespread.

distribution of osteochondromas originating from the metaphyses of long bones.

**Case presentation:**

This case study examines a 55-year-old male cadaver bequeathed to the University of Liverpool who suffered from HME, thus providing an exceptionally rare opportunity to examine the anatomical changes associated with this condition.

**Conclusions:**

Findings from imaging and dissection indicated that this was a severe case of HME in terms of the quantity and distribution of the osteochondromas and the number of synostoses present. In addition, the existence of enchondromas and the appearance of gaps within the trabeculae of affected bones make this a remarkable case. This study provides a comprehensive overview of the morbidity of the disease as well as adding to the growing evidence that diseases concerning benign cartilaginous tumours may be part of a spectrum rather than distinct entities.

## Background

Hereditary multiple exostoses (HME) is characterised by the presence of multiple osteochondromas situated throughout the skeleton in bones that form by endochondral ossification. Osteochondroma formation is caused by abnormal chondrocyte proliferation in the growth plate which subsequently herniate through the adjacent periosteum [1]. These outgrowths undergo ossification to create benign tumours with a cortex and medulla that are continuous with the underlying bone. There are two classifications of osteochondroma defined by their attachment to the bone; a broad base attachment is termed sessile while a slim attachment with an expanded head is called pedunculated [2]. The surface of an osteochondroma is coated by a cartilage cap that is derived from, and acts as, the epiphyseal growth plate. The growths originate from the metaphysis but the larger tumours can also expand around the diaphysis or the epiphysis. The exostoses in HME are also characterised by their growth away from the neighbouring epiphysis [2].

Osteochondromas account for 20–50% of all benign bone tumours [3] equating to 10% of all bone growths [4]. They commonly appear a singular entity with only 10% of patients suffering from the multiple form seen in HME [1, 5]. The disease has a prevalence of between 1:50,000 and 1:100,000 [3]. The complications of the disorder can be severe and vary greatly between individuals reflecting differences of osteochondroma distribution and size. A common theme however is the discomfort experienced by sufferers with 60% of children and 80% of adults experiencing chronic pain [6].

The exostoses cause long bones to undergo bowing, undertubulation and synostoses if growths appear on adjacent bones. Overall, this can affect height with 40% of patients being of short stature [7]. Locally the larger growths can physically compromise the surrounding anatomy causing damage to soft tissues. Notably this can cause entrapment and subsequent rupture of tendons and the formation of bursae over areas of constant friction [8]. In addition, osteochondromas can lead to vascular compromise [9], nerve compression with ensuing denervation of muscles [10] and extrinsic pressure erosion of neighbouring bones [7]. In the case of pedunculated growths their thin stalks make them more prone to fracture [4].

HME is an autosomal dominant disease caused by mutations and subsequent loss of function in exostosin (EXT) genes [6]. Penetrance of 100% is observed in males whilst it is slightly less in females at 96% [11]. EXT-1 mutations have been shown to result in more severe phenotypes with a higher numbers of exostoses, an increased incidence of osteochondroma growth in flat bones and most notably an increased incidence of malignant change to chondrosarcomas [11].

The reported rate of malignant change in sufferers of HME does vary, however most accounts have it between 0.5–5% [4, 12–15] with the average age stated as 30 years old [15]. The malignancies formed are 9 times more likely to be a chondrosarcomas formed in the cartilage cap than an osteosarcoma [4]. In normal osteochondroma progression the cap degenerates to become thinner in the adult, as is normal for any growth plate. Therefore, a cap width of more than 1.5 cm in the adult should be viewed with suspicion [7].

With such a rare disease the majority of evidence has been inferred from imaging studies of living patients. This study is the first to correlate the radiological signs with a targeted and detailed anatomical investigation in adulthood, thus providing a more complete view of HME and informing future patient studies.

## Case presentation

A 55-year-old male cadaver who had suffered from HME was donated to the University of Liverpool for anatomical investigation. Consent was given by the donor ante-mortem and ethical approval for the study was obtained via the Health and Life Sciences Committee on Research Ethics. The cadaver was embalmed four days post-mortem with CT scans and radiographs obtained to establish a complete record of the cadaver. Following dissection bones were removed for MRI and microscopic investigation. The radius, ulna, femur, tibia and fibula were isolated and scanned in a 1.5 T Siemens Symphony MRI to document soft-tissue features. Multiple modalities were used with T2 turbo spin echo (TSE) and standard dual echo steady state (DESS) sequences [16] chosen for this publication because they provided the best combination of contrast and spatial resolution. Following this the bones were sectioned to observe the internal anatomy and samples were removed to examine histologically. These sections were embedded in a methyl methacrylate resin and sectioned for mounting on slides. Following deplasticising with methoxyethyl acetate the sections were stained with 1% Toluidine Blue for 10 min at room temperature (please refer to [17] for full method). Recovery of DNA from cells was not possible due to the nature of formalin fixation.

### Full body scans

A combination of pre-dissection CT scans and radiographs showed that the cadaver had at least 73 separate external skeletal defects ranging in size and form and were apparent throughout the skeleton. The lower limb contained the most exostoses with 34 tumours, 5 of which were in the feet. The vertebrae displayed 22 growths with 12 located on the vertebral bodies and 10 on the spinous and transverse processes. Six growths were noted in the upper limb with 4 located on the scapula. Two exostoses were on the posterior iliac crests and 2 were located on the anterior of the sternum. The 7th left rib had a small growth on the sternal end, while the right 8th and 10th left rib had osteochondromas on the tubercles. No major dysplasia was evident in the bones of the skull, including the cranial base.

On the basis of the features observed in the CT scans and radiographs the following dissection was targeted to the right elbow and right lower limb.

### Elbow

**Fig. 1:**
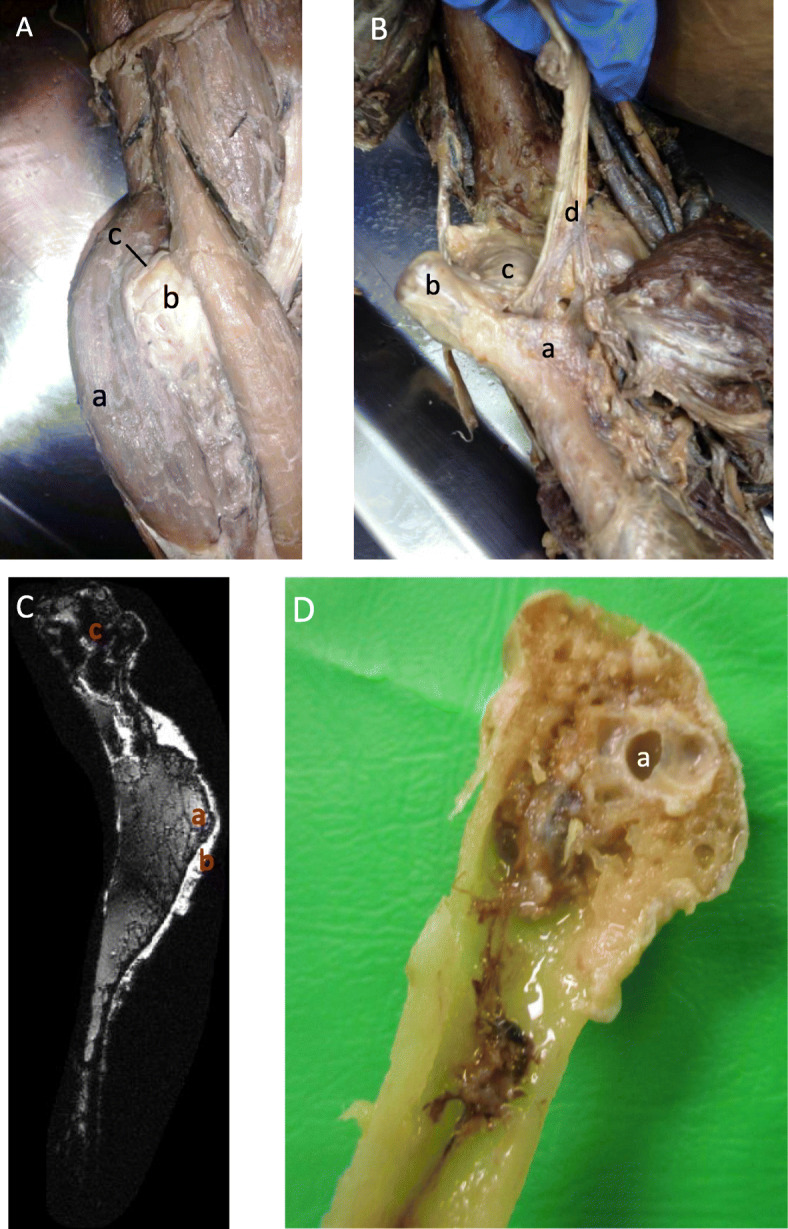
Right proximal forearm. **a** Superficial dissection. A tumour has caused lateral displacement of the brachioradialis muscle (**a**). The radial nerve (**c**) has been diverted over the superior surface of radial head (**b**). **b** Deep dissection. Exostosis in the region of the radial tuberosity (**a**), radial head dislocation (**b**). The capitulum (**c**) formed an articulation with the radius at the radial tuberosity (**a**), causing the biceps brachii tendon (**d**) to be incorporated into the joint. **c** DESS MRI of proximal radius. An osteochondroma present at the radial tuberosity (**a**) has a continuous cortex and medulla with the underlying bone. A cartilage cap is present on the surface (**b**). Note the low signal within the head of the radius (c). **d** Sagittal section of radial head. Radius displays a large void (**a**) within the epiphysis

Bowing of the radius caused by a shorter ulna had resulted in a radial head dislocation from the elbow joint. Dissection of this area revealed the extent of the soft tissue disfigurement with brachioradialis displaced laterally around the radial head while the radial nerve was stretched over its superior surface, quite possibly causing nerve entrapment (Fig. 1a).

The osteochondroma in the metaphysis of the radius had caused an unusual articulation at the elbow joint with the capitulum of the humerus now articulating with the deformed radial tuberosity (Fig. 1b). Interestingly the biceps brachii tendon had been incorporated into the joint itself.

MRI scans of the proximal radius reveal the cartilage capped exostosis forming at the radial tuberosity (Fig. 1c) and the image also shows large signal voids within the trabeculae that were devoid of bone during further dissection (Fig. 1d).

### Proximal femur

**Fig. 2:**
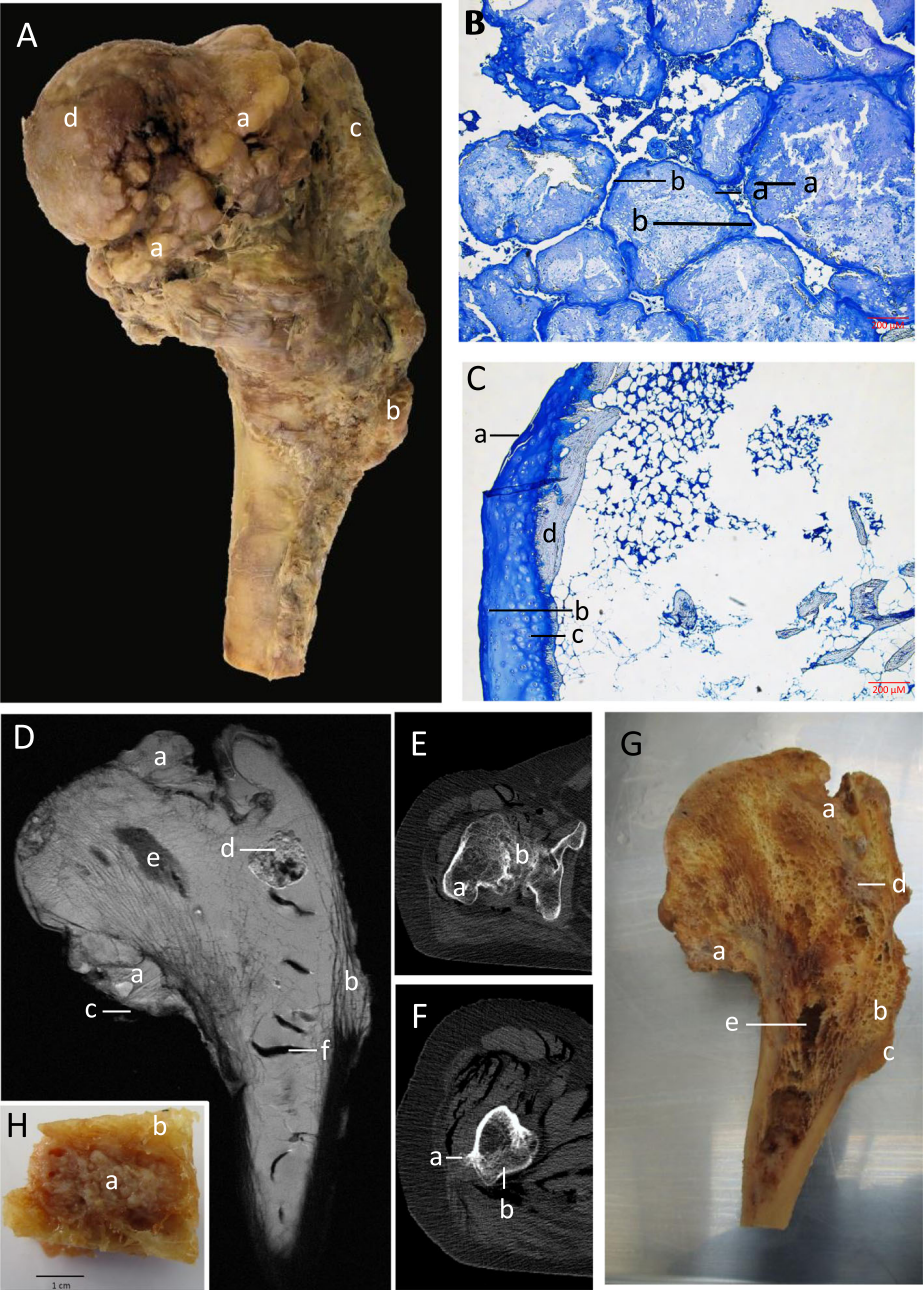
Right proximal femur. **a** Posterior view. A large exostosis (**a**) surrounding the metaphysis with an outgrowth (**b**) from the lateral portion of the femoral shaft. Greater trochanter (**c**) and femoral head also displayed (**d**). **b** A section through intramedullary mass. (**a**) Cartilage matrix (**b**) inter nodular cartilage. **c** A section through the surface of a sessile osteochondroma. (**a**) Perichondrium on surface of cartilage cap. (**b**) chondrocytes. (**c**) Hypertrophic mature chondrocytes. (**d**) Subchondral bone. **d** T2 MRI coronal section. Osteochondromas can be seen surrounding the metaphysis and extending from the diaphysis (**a** and **b** respectively). Cartilage caps can also be seen on the osteochondromas (**c**). A large fluid filled mass is present in the metaphysis (**d**). Large voids are present in both the epiphysis and diaphysis (**e** and **f**). **e** Transverse CT scan through greater trochanter (**a**). Showing osteochondroma (**b**). **f** Transverse CT scan through proximal diaphysis. The pedunculated osteochondroma (**a**) can be seen on the lateral surface whilst the sessile growth can be seen on the posterior diaphysis (**b**). **g** Coronal section. (**a**) Cross section of osteochondroma. (**b**) trabecular bone obliterating normal cortical bone as the medulla of the overlying exostosis. (**c**) Cortex of exostosis. (**d**) Soft mass located in the metaphysis. (**e**) An example of the gaps located within the trabecular bone. **h** Cartilaginous mass removed (**a**) surrounded by trabeculae (**b**)

The proximal femur had 3 exostoses on its surface, varying greatly in size and shape. The largest was formed around the medial and anterior aspects of the metaphysis; it was sessile, had a large amount of lobulation and was capped in cartilage. This large exostosis completely obliterated the neck of the femur causing difficulty in discerning the greater and lesser trochanters of the femur (Fig. 2a and e). Inferiorly there was a small pedunculated growth on the lateral aspect of the shaft. Adjacent to this, inferior to the greater trochanter, was a long sessile exostosis that extended towards the diaphysis (Fig. 2a and f).

MRI scans showed osteochondromas surrounding and growing from the metaphysis (Fig. 2d) with cartilage caps present on their surface. The scans also revealed a mass within the metaphysis; given the location of this growth it was suspected to be either an enchondroma or a chondrosarcoma. Interestingly, similar to the head of the radius in the previous section, the MRI scan revealed large signal voids within the entire proximal femur.

The histological section through the pedunculated growth (Fig. 2c) showed a cartilage cap with underlying subchondral bone. The cap showed distinct features of a growth plate including a perichondrium and mature and immature chondrocytes.

To further discern the anatomy of the internal structures the proximal femur was bisected (Fig. 2g). A soft mass (Fig. 2h) corresponded to the area of high intensity on the T2 MRI scan. A sample was taken for histological study and showed a large cartilage mass organised into discrete areas, creating a lobulation effect (Fig. 2b). The dark areas noted in the MRI corresponded to large voids within the trabecula bone.

### Tibia and fibula

**Fig. 3:**
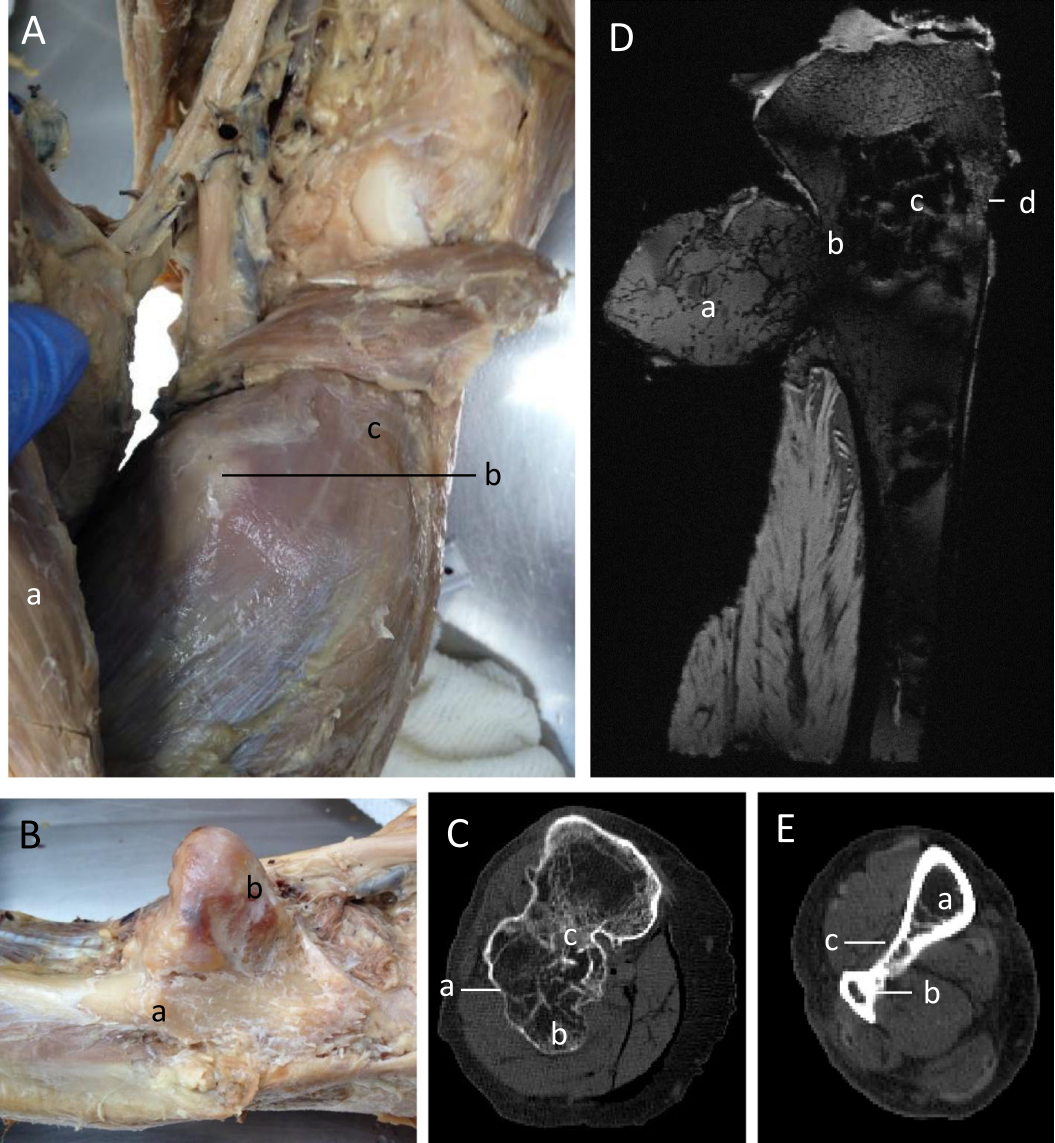
Right proximal leg. **a** Superficial dissection. Gastrocnemius (**a**) has been reflected to show the osteochondroma (**b**) of the head of the fibula causing deformity of the soleus muscle (**c**). **b** Lateral view. (**a**) Fibula. (**b**) Cartilage capped osteochondromas of head of fibula. **c** Transverse CT scan through fibular head. Osteochondroma visible with cortical bone (**a**) and trabecular (**b**) continuous with the underlying normal bone. A synostosis between the tibia and fibula can be seen (**c**). **d** DESS MRI sagittal section. The osteochondroma of the fibular head can be seen containing trabecular bone (**a**). The synostosis between the fibular and tibia heads can be observed (**b**). Like the femur and radius the tibia contains large gaps in the trabecular bone structure (**c**). Cortical destruction has occurred (**d**). **e** Transverse CT scan through distal tibia and fibula. An osteochondroma on either the tibia (**a**) or fibular (**b**) has formed a synostosis (**c**) at the distal end of the two bones

Like the radius, the exostosis in the head of the fibular was a large, lobulated mass with a cartilage cap and had caused considerable anatomical changes to the surrounding soft tissue. By extending into the muscle belly of soleus it had stretched and torn the muscle fibres (Fig. 3a and b).

CT scans of the growth revealed the full extent of the osteochondroma and showed that a synostosis had formed between the fibular and tibia heads (Fig. 3c). Like the previous scans of the radius and femur the DESS MRI scans of the tibia revealed large signal voids within the trabecular bone structure. This may have compromised the overlying bone as the cortex appeared thinner in this area (Fig. 3d). It should be noted that a synostosis had also occurred at the inferior tibiofibular joint (Fig. 3e).

### Feet

**Fig. 4:**
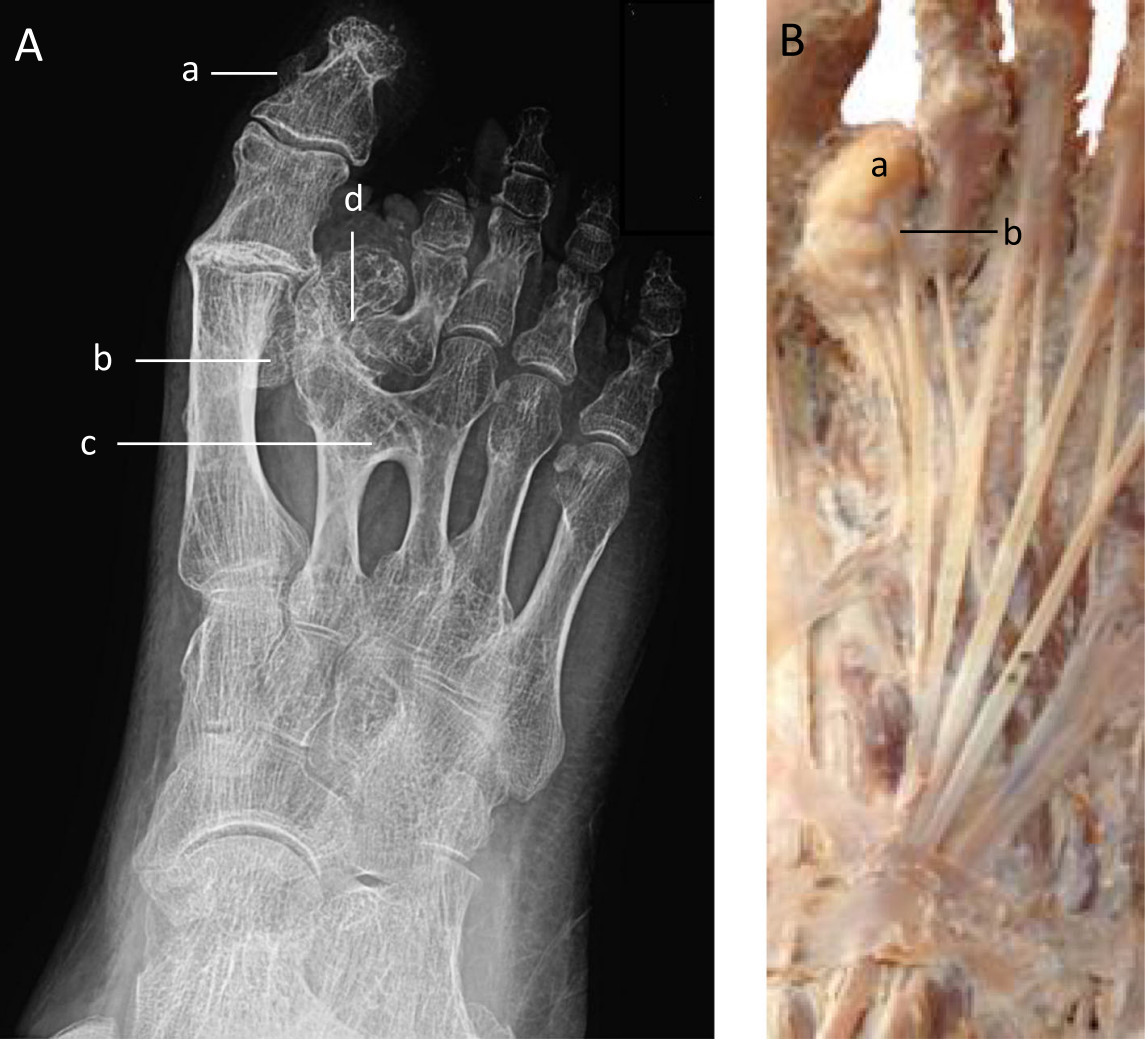
Right foot. **a** Radiograph. A pedunculated growth is present on the medial surface of the 1st distal phalanx (**a**). A sessile osteochondroma is situated on the inferior surface of the 1st metatarsal bone (**b**). A sysnostosis has formed between the heads of the 2nd and 3rd metatarsals (**c**). An osteochondroma of the 2nd metatarsal has altered the orientation of the metatarsophalangeal joint (**d**). **b** Superficial dissection. The cartilage capped osteochondroma can be seen at the head on the 2nd metatarsal (**a**). A tendon of extensor digitorum longus is observed inserting into the osteochondroma (**b**)

The right hallux had a pedunculated growth on the medial aspect of the distal phalanx. There was also an exostosis present on the inferior surface of the head on the 1st metatarsal (Fig. 4a).

The anomaly that gave the external appearance that the second digit was shorter is in fact due to a growth of the 2nd metatarsal. The head of the metatarsal had become so overgrown that it had displaced the proximal phalanx of the 2nd digit laterally causing the articulation between the two to be in a sagittal plane as opposed to the normal coronal facing joint (Fig. 4a and b). Furthermore, a synostosis had occurred between the heads of the 2nd and 3rd metatarsal bones (Fig. 4a). The dissection of this area revealed the cartilage capped osteochondroma had a tendon of extensor digitorum longus unusually inserting into it (Fig. 4b).

## Discussion and conclusion

Findings reported here suggest that this is a severe case of HME with some interesting features. The severity of HME does vary between individuals and while no consensus has been reached regarding classification, Mordenti et al. [18] have devised a simple class system based on the deformities and functional limitations of sufferers in order to provide clarity between cases. Class I has no deformities and no functional limitations; class II has deformities and no functional limitations while class III has both deformities and functional limitations. Each class is further subdivided into A and B depending on severity. This case study clearly fits into class IIIB as there are deformities present and functional limitations in more than one anatomical area.

The tumours were widespread; exostoses could be seen in the scapulae, ribs, humeri, radii, ulnas, pelvis, femurs, tibias, fibulas, feet and vertebrae. Whilst there are no signs of growths within the hands the medical notes state that tumours in the proximal phalanx of the right 2nd digit were surgically excised 25 years ante-mortem. The literature reports the most affected parts of the skeleton are, in order, the long bones, the scapulae, the ribs, the pelvis and the vertebrae [6].

The most common site of osteochondromas in HME is the knee, with reports as high as 94% of sufferers having a knee exostosis [19]. These can either be in the distal femur which occurs 90% of the time [2], the proximal tibia or proximal fibula which have osteochondromas in 84 and 76% of HME cases respectively [2]. A third of HME sufferers report genu valgum due to the deformities present at the knee [6]. As previously noted, this case has osteochondromas in all 3 locations on both lower limbs.

Deformities of the forearm are quite common in HME sufferers with 30–60% of patients suffering from them [20]. They can often lead to poor elbow function and chronic pain [21]. The proximal radius and ulna are involved in 37 and 38% of cases respectively while an exostosis in the distal ulna has been reported in 80% of cases [19]. These forearm deformities have recently been reclassified into 4 groups by Jo et al. [22] from the long standing 3 categories proposed by Masada et al. [23]. Class I comprising an exostosis at the distal ulna with subsequent shortening and bowing of the radius. Class II are deformities with radial head dislocations; IIa with an osteochondroma in the proximal radius, as in this case, and class IIb without. Class III are forearms with a distal exostosis in the radius and shorter radius and class IV forearms have exostoses in the distal radius and ulna.

It is the exostosis at the distal growth plate of the ulna that ultimately leads to dislocation of the radial head. The distal growth plate of the ulna has a smaller cross section than the equivalent in the radius and therefore greater proportional contribution to growth of the forearm. An exostosis here disrupts ulna growth and subsequently forces the radius to undergo bowing to compensate. It is this bowing that leads to subluxation or full dislocation from the elbow joint [20, 24]. Dissection allowed for a close examination of how this affected the elbow joint. Interestingly the capitulum of the humerus had formed a new joint with the exostosis formed on the radial tuberosity with the cartilage cap appearing to act as the articular cartilage. Furthermore, the tendon of biceps brachii was incorporated into the joint between the two bones and therefore may have acted like a pseudo articular disc (Fig. 2b). As well as the pain that may have been experienced with a tendon acting between two bones the supination function of biceps brachii must have been severely affected with the change in orientation of the insertion of its tendon. This relocation of the biceps brachii tendon has not previously been described. The radial nerve, alongside the common fibular nerve, are the most likely to suffer from nerve entrapment [6] in HME patients. This dissection allowed a greater view of how this may occur with the radial nerve stretched over the head of the radius and may indicate another reason to act surgically upon HME patients with elbow dislocations.

Four exostoses where found in the scapulae of donor. 3–4.6% of all osteochondromas are found in this bone with the ventral surface more commonly affected than the dorsal surface [25]. However, in this case both scapulae had a ventral and dorsal osteochondroma each. The consequences of exostoses in the scapulae can range from crepitus, pseudo winging of the scapula and weakness with reduced muscle mass of the pectoral girdle muscles [25]. Having an exostosis in the shoulder is an indication for an increased rate of malignant change with some reporting as high as 43%, however, this is likely due to both being associated with mutations in EXT-1 [19].

Exostoses in ribs only make up 1% of all growths reported in HME, in this case, however, they make up 4% of all tumours. The 1% reported is likely a gross underestimation as only symptomatic tumours are reported. Indeed Mazza et al. [26] found asymptomatic tumours in the ribs during investigations into symptomatic growths. Rib exostoses can be dangerous given their location to thoracic organs, complications include spontaneous haemothorax, pneumothorax and extrinsic coronary compression [19]. This case also details the presence of a sternal osteochondroma which, to our knowledge, have not been previously described.

Cases of HME with vertebral lesions have commonly been reported between 1 and 9% [5, 27], however more recently some reports have reported incidences of 23% [27], 38% [28] and 68% [2, 27]. Whilst some of these studies may be overestimating due to selection bias it is clear that examining all HME cases for vertebral lesions, and not just symptomatic cases, leads to higher reporting of incidence rates. In addition, it has been stated that in-vivo radiographs are poor at ascertaining the presence of vertebral lesions [28]. Therefore, screening of all HME patients is more likely to give an accurate account of the burden of the disease in the spine.

Lesions of the vertebral column are more common in the superior spine; 30–80% of the lesions have been reported in the cervical spine, 20–30% in the thoracic spine and only a few reports have cases in the lumbar region [29]. Specially C2 is the most common vertebra to have a tumour followed by C3 and C6 [27]. Jackson et al. [28] reported that the average lesions in a single patient was 2.25 with the range being between 1 and 4, in addition it has been stated that finding lesions in multiple levels of the vertebral column is rare [29]. The osteochondromas that do grow on the vertebrae can occur in multiple areas reflecting the many ossifications centres; they are found on the vertebral bodies, tips of the spinous processes, the transverse processes and the articular processes [5]. Intracanal lesions are of great concern as they can compress the spinal cord. The reported incidence of these tumours range between 4 and 27% of cases [30] and are mostly located in the cervical region [5].This study reports 22 lesions throughout all levels of the vertebral column, they are located on the vertebral bodies, the spinous processes and the transverse processes with no obvious cases of intracanal lesions on imaging. Whether the high number of lesions indicate a more robust study that is more representative of the disease or whether this is an extreme case of HME remains to be seen and will only be answered with similar full body investigations.

Synostosis of neighbouring bones has previously been described in osteochondroma sufferers [31]. This event occurs when two osteochondromas grow on neighbouring bones and then fuse together. Bessler et al. [31] presented a study of 21 HME sufferers and reported synostoses in 10 of them. Two of these patients had 4 synostoses all located in the lower limb [31]. Synostoses are unlikely to form in the forearm due to the movement that occurs between the radius and ulna. This case presented with 5 different regions where bones had fused together: the right tibia and fibula, both proximally and distally, the proximal left tibia and fibula and the 2nd and 3rd metatarsal bones on both feet.

One of the most significant findings from this case study is the presence of cartilage masses within the metaphyses of the femoral heads which are likely to be either enchondromas or chondrosarcomas. Enchondromas are benign cartilaginous rests that continue to grow but have been displaced from the growth plate [32]. Where the chondrocytes in an osteochondroma herniate through the periosteum to grow on the surface of the bone an enchondroma remains intramedullary. While it is known that differentiating between an enchondroma and a grade 1 chondrosarcoma is difficult [33], a chondrosarcoma is usually larger than 3 cm [34], causes scalloping of the cortex [32, 34] and leads to entrapment of the surrounding boney trabeculae [32, 33]. Histologically a hypercellular mass may indicate a chondrosarcoma, however in diseases of multiple enchondromas (Ollier’s disease) this is not the case [33]. Enchondromas are also described as being nodular with fibrous septa in between [33]. These features strongly suggest that the mass in the right femoral head is an enchondroma or, at the very least, is a secondary chondrosarcoma derived from an existing enchondroma. The mass in the left femoral head is more indicative of a secondary chondrosarcoma due to its size and invasion of the cortex.

The presence of enchondromas alongside osteochondromas raises the differential diagnosis of metachondromatosis. However, this is unlikely to be the case here as the location, anatomy, severity, deformities and disease progression of the osteochondromas are all classic signs of HME. The osteochondromas present in metachondromatosis are predominantly found in the hands and feet [3, 35, 36] and have been reported to be symmetrical [37]. While this case had some lesions of the hands and feet the widespread distribution throughout the majority of the skeleton and the severity of these large osteochondromas have never been reported in cases of metachondromatosis. A critical sign of osteochondromas in metachondromatosis is that they point towards the nearest epiphysis [36–40]. Here we report classic HME type pedunculated lesions that point away from the joint. Periarticular calcifications are commonly present in metachondromatosis however none were found in this investigation [36, 38, 40]. Metachondromatosis patients have never reported subluxation or dislocation of the radial head which again is closely associated with HME [3, 35, 38, 41]. In addition, to our knowledge, no synostoses have ever been reported in a case of metachondromatosis. Finally, in cases with a complete follow up to adulthood, the exostoses in metachondromatosis regress post adolescence [38, 40]. Again, this is clearly not the case in this study.

MHE and metachondromatosis are both conditions of improper chondrocyte proliferation. Recent studies have suggested that despite having distinct genetic profiles there may be overlap between these conditions. Vining et al. [42] reported a family displaying symptoms of both metachondromatosis and HME with the father showing a mutation in the EXT-2 gene. Goud et al. [34] examined 7 HME patients with either EXT-1 or EXT-2 mutations who had cartilaginous masses within bone. Although it was concluded that these were chondrosarcomas there was strong evidence to suggest that they had come from existing enchondromas. On the opposite side Kanaya et al. [41] reported a case of metachondromatosis that had no enchondromas present. All three studies come to the same conclusion that the differences between these diseases may be gradual rather than absolute. The present study, although only a single case, adds to this small but growing number of findings that suggest a closer link between these conditions of chondrocyte proliferation.

A search of the literature reveals no information concerning the voids within the trabeculae that were seen in the femoral head, the tibia and the radius. Given the locations of these spaces it is possible that they are connected to the disease. There is evidence that shows the regression of enchondromas and transformation to bone marrow in adults [43]. Although unusual, this may explain the areas of non-trabecula bone seen in the cadaver. Future studies that closely examine this condition should look for these to determine if any patterns exist.

In conclusion this case study represents the most detailed anatomical investigation into HME to date. By collecting both imaging and dissection data it has revealed a more complete picture of HME presentation, detailing a widespread distribution of osteochondromas, especially in the vertebrae, and a high volume of synostoses. This case study also documents trabecular voids and the presence of enchondromas in the femoral heads. These warrant closer scrutiny in future patient studies. As body donation increases, it is possible that more HME suffers will come forward for detailed anatomical investigation; this may reveal how common these findings are and may aid treatment of the condition in the future.

## Data Availability

All relevant data is included in manuscript.

## Abbreviations

CT: Computed tomography
HME: Hereditary multiple exostoses
MRI: Magnetic resonance imaging
DESS: Dual echo steady state.
TSE: T2 turbo spin echo
